# Supplementary material for: Balancing Performance and Human Autonomy With Implicit Guidance Agent
Source: Front Artif Intell. 2021 Sep 21;4:736321. doi: 10.3389/frai.2021.736321 (PMC8490733; doi:10.3389/frai.2021.736321)
Supplement: Supplementary file 1 [file Presentation1.pdf]

# Supplementary Material

## 1 EXPERIMENTAL TASKS

All the concrete tasks performed by participants are shown below. Red objects are the best target, and the color is blue same as the other object in the experiments except for the task with the explicit guidance agent.

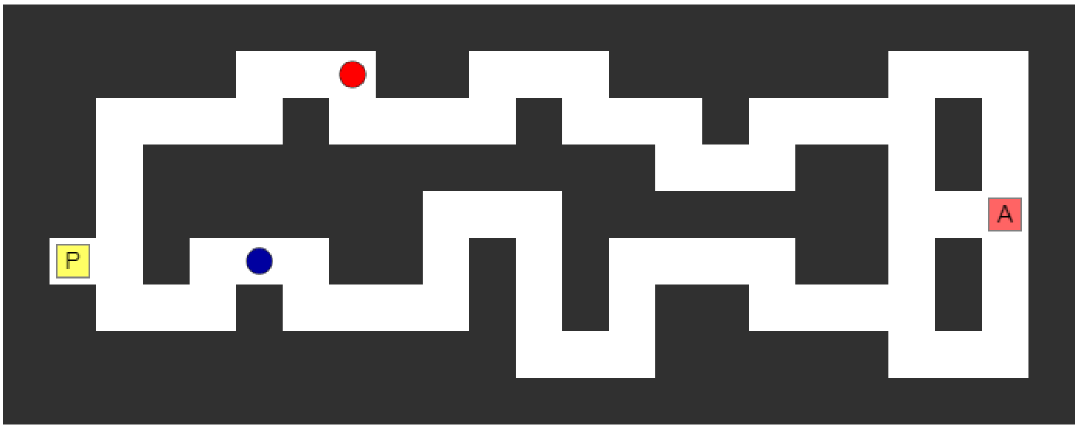


**Figure S1.** Task 1

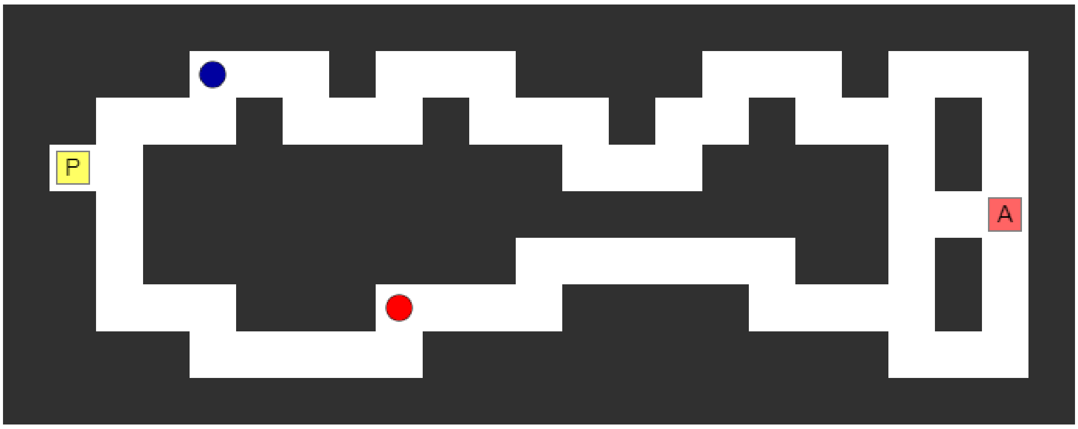


**Figure S2.** Task 2

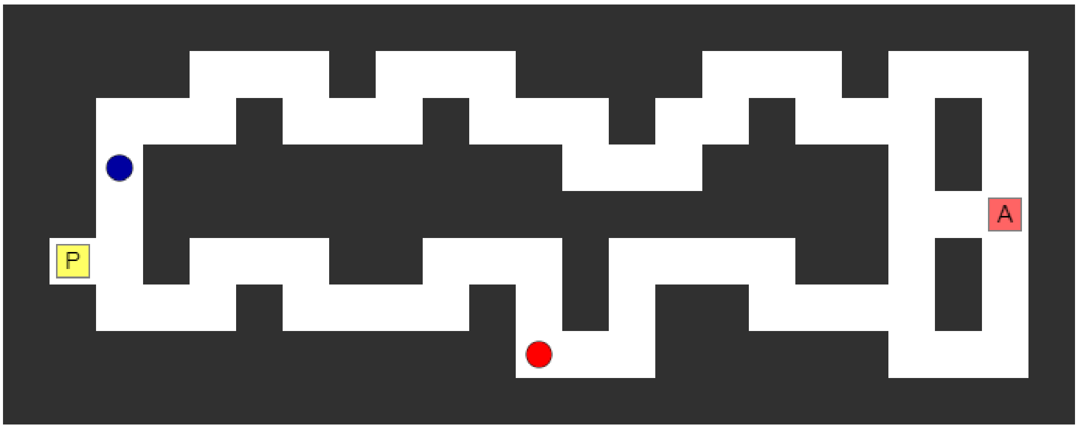


**Figure S3.** Task 3

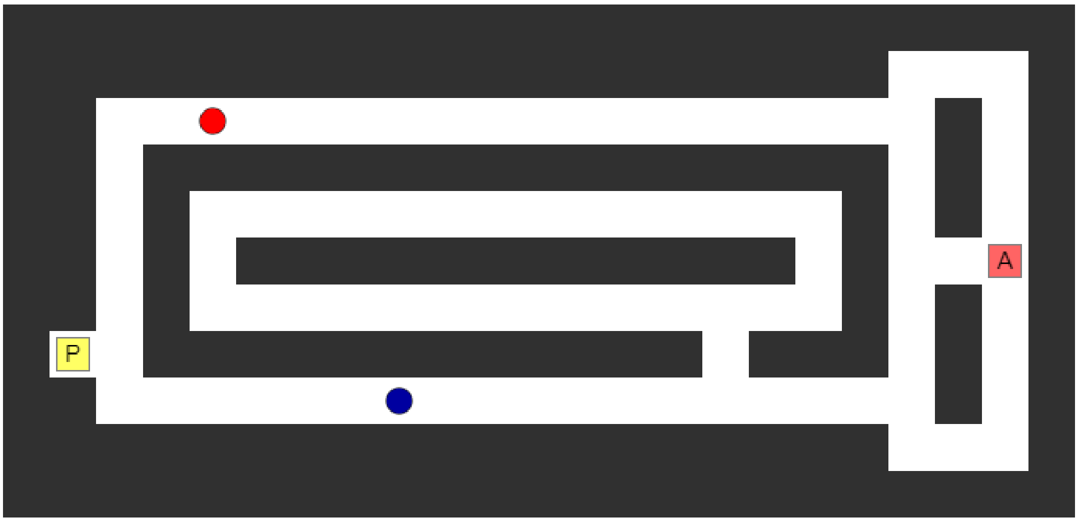


**Figure S4.** Task 4

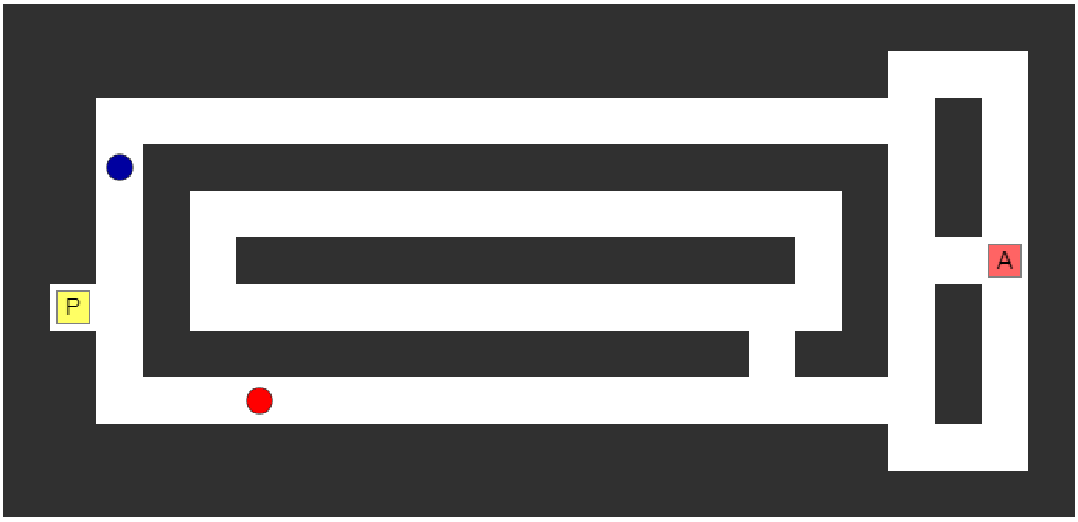


**Figure S5.** Task 5

## 2 EXPERIMENTAL DATA

We attached the data of experimental results in a CSV format. The file “table 1.csv” is the results of the captured target in the task. The file “table 2.csv” is the results of the survey.
